# Biochemical characterization and pharmacognostic evaluation of purified catechins in green tea (*Camellia sinensis*) cultivars of India

**DOI:** 10.1007/s13205-014-0230-0

**Published:** 2014-06-14

**Authors:** Jigisha Anand, Bhagwati Upadhyaya, Pramod Rawat, Nishant Rai

**Affiliations:** 1 Department of Biotechnology, Graphic Era University, Dehradun, India; 2 Department of Microbiology, Guru Nanak Dev University, Amritsar, Punjab India

**Keywords:** Phytoconstituents, GT, *Camellia sinensis*, Flavonoid, HPLC

## Abstract

Green tea (GT) is derived from the leaves of *Camellia sinensis* implicated in a wide range of health attributes. In the present comprehensive study, methanolic, acetone and aqueous extract of leaves of *C. sinensis* var. *sinensis* [Kashmir (KW), Uttarakhand (IP & PN)] and *C. sinensis* var. *assamica* (Assam, AT) were explored for their phytoconstituents. Solvent extracts of GT cultivars showed rich presence of phytoconstituents in comparison with aqueous extracts. The methanolic extract of AT and acetone extract of KW showed highest total phenol content (18.32 ± 0.357 mg of GAE equivalent/g of sample) and total flavonoid content (29.25 ± 0.015 mg of catechin equivalent/g of sample), respectively. All the cultivars revealed higher free radical scavenging activity in the range of 73.80 ± 0.152 to 82.40 ± 0.004 % confirming antioxidant potentials. The HPLC analysis of purified residue procured from solvent partitioning depicted AT with highest concentration of epigallocatechin gallate (EGCg) i.e., 154.7 ± 4.949 mg/g followed by Kashmir and Uttarakhand GT cultivars. The present study revealed that Assam GT could be a potent herbal candidate with multiple nutraceutical applications. However, significant investigation of the cultivars is to be done to further explore the EGCg-dependent activity of GT for herbal drug development.

## Introduction

In recent years, nutritional therapy and phytotherapy have emerged as new concepts of health aid. Plant-derived nutraceutical or functional foods have received considerable attention because of their presumed safety and potential nutritional and therapeutic effects. Nutraceuticals serve essentially as therapeutics on patients suffering with several debilitating diseases, and are useful as health giving food supplements for general population (Pandey et al. 2011).

Green tea in its purest and most unadulterated form has always influenced human health from generations, and day-by-day scientific evidences throughout the world are making people aware of health benefits associated with this herbal drink. It is obtained from dried leaves of *Camellia sinensis* (L.) Kuntze, which is an angiosperm dicot plant (Anand et al. 2012). Commercial tea cultivars are recognized under three different taxa, namely, *C. sinensis*, *C. assamica*, and *C. assamica* ssp*. lasiocalyx* (Baruah 1965). However, tea is highly heterogeneous (Gulati et al. 2009), and all the above taxa freely inter-breed, resulting in a cline extending from extreme China types to those of Assam origin (Wight 1962).

There are two main varietals of *C. sinensis*, *C. sinensis* var. *sinensis*, better known as China bush, and *C. sinensis* var. *assamica*, also known as Assam bush. However, altogether, there are more than a thousand sub varieties of *C. sinensis*.

**Fig. 1:**
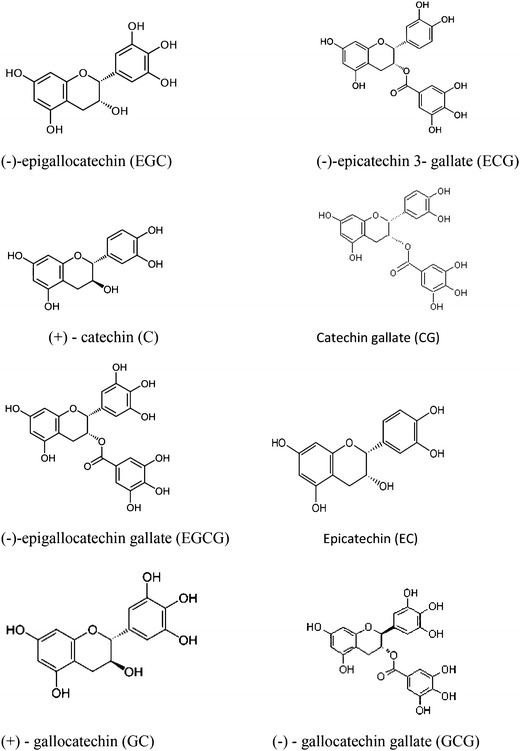
Structures of different catechins present in green tea

Phytochemicals are the bioactive compounds that occur naturally in plants. They include secondary metabolites, many of which are synthesized for plant defences and adaptation to environmental stress (Mcclanahan 2012). Tea is reported to contain nearly 4,000 bioactive compounds of which one-third is contributed by polyphenols (Tariq et al. 2010). The bioactive compounds present in GT leaves are alkaloids, flavonoids, steroids, phenols and terpenoids, which serve as valuable starting material for the medicine development (Lister and Wilson 2001). Among the natural polyphenols present actively in GT are the flavonoids commonly known as catechins. The flavonoids (and their fraction, catechins) are the basic phenolic compounds in green tea responsible for antioxidant activities such as neutralization of free radicals that are formed in the process of metabolism (Horzic et al. 2009). Some major catechins are (−)-epigallocatechin gallate (EGCg), (−)-epigallocatechin (EGC), (−)-epicatechin 3-gallate (ECG) and epicatechin (EC). Other minor catechins like catechin gallate (CG), (+)-gallocatechin (GC), (−)-gallocatechin gallate (GCG) and (+)-catechin (C) (Fig. 1.) are also present in tea (Yamamoto et al. 1997).

The EGCg, are the major catechin in GT which is believed to be the primary source of green tea’s beneficial effects (Fujimura et al. 2002). EGCg have shown to be promising in promotion of cardio-vascular health, cancer prevention, and skin protection, to fight high cholesterol levels, infection, impaired immune function, diarrhoea, and fatigue. The most notable health benefit of GT is its powerful antioxidant potential which, at the molecular level, help prevent cellular damage from certain oxidation reactions in the body (Anand et al. 2012). The higher antioxidant activity of green tea makes it more beneficial in protecting the body from oxidative damage due to free radicals. It appeared that these antioxidants slow or halt the initiation of cancer, heart disease, suppress immune function, and accelerated aging (Hamilton-Miller 2001).

Although the catechins in tea leaves were identified long ago, the regulatory mechanism governing catechin biosynthesis remains unclear (Xiong et al. 2013). The genetic differences between the hybrids are well reflected in biochemical composition of leaves. However, biochemical composition, as varied between varieties, is yet to be fully utilized in tea taxonomy (Sanderson and Kanapathipillai 1964). It has been studied that the cultivars differ in their antimicrobial potentials, which is correlated with their inherent catechin composition. Therefore, we can say that there is a differential gene expression in GT cultivars with different morphology and catechin content (Yang et al. 2012).

The regional variation of quality within the tea growing region can be attributed to genetic diversity and its interaction with the environment (Sabhapondit et al. 2012). Environmental factors such as pedoclimatic (soil type, sun exposure, rainfall) or agronomic (culture in greenhouses or fields, biological culture, hydroponic culture, fruit yield per tree, etc.) have a crucial role in the composition of catechins (Manach et al. 2004). To ascertain diversity, careful study of secondary metabolites, especially those which are major contributors to quality, is essential. Total catechin content could be used to indicate the quality potential of tea, with high content being related to high quality (Obanda et al. 1997).

In our study, the finding suggests vast regional variation among the catechin composition of purified residues of leaves of GT cultivars collected from different regions of Indian subcontinent. Also, much variation on the quantitative analysis of different solvent extracts has been observed.

## Materials and methods

### Collection of the samples

**Fig. 2:**
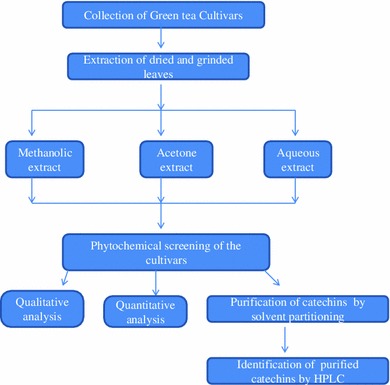
Flow diagram showing phytochemical and pharmacognostic study of GT cultivars

Green tea leaves from Assam (AT), Kashmir (KW) and Uttarakhand region (IP and PN) of North India were collected and identified by Dr. S. K. Srivastava, Scientist ‘D’ from Botanical Survey of India, Dehradun. The collected leaves were washed thoroughly under fresh water and left for drying under shed for 2 weeks. The dried leaves were then powdered in a blender and kept in sealed packets in refrigerator at 4 °C until further use. The methodology followed in the present study is shown as flow diagram (Fig. 2).

### Preparation of tea extract

For the preparation of solvent extracts, dried powdered leaves were separately soaked in 70 % methanol and 70 % acetone in ratio plant: solvent (1:10) and allowed to macerate for consecutive 2 days. After maceration, the extract was filtered using Whatman No. 1 paper and the solvents were completely evaporated at appropriate temperature till it gave a viscous mass. The crude extract was weighed and its percentage yield was recorded (Table 2). The crude extract was stored at 4 °C until further use (Archana and Abraham 2011).

Aqueous extraction of the leaves samples was done by soaking the leaves in sterilized distilled water at approximately 1:10 w/v ratio for 1 h. After soaking, the decoction was brought to boil for 60 min. The liquid extract was separated from the solids by filtration with double-layered muslin cloth and allowed to precipitate. The crude extract was weighed and its percentage yield was recorded (Table 1). The crude extract was stored at 4 °C until further use (Koh et al. 2009).

**Table 1:** Qualitative estimation of phytochemicals in aqueous and solvent extracts of different green tea cultivars

Phytochemicals	Methanolic extract				Acetone extract				Aqueous extract			
	AT	KW	IP	PN	AT	KW	IP	PN	AT	KW	IP	PN
Phytosterol	++	++	++	++	+++	+++	++	++	+	+	+	+
Carbohydrates	+++	+++	+++	+++	++	+++	+++	++	+	+	−	–
Saponin	–	–	–	–	–	–	–	–	–	–	–	–
Catecholic Tannins	+++	+++	+++	+++	+++	+++	+++	++	–	–	–	–
Alkaloids	+	+	+	+	+	+	+	+	+	+	+	+
Flavonoids	+++	++	++	++	+++	+++	+++	+	+	+	+	+
Anthocyanins	–	–	–	–	–	–	–	–	–	–	–	–
Proteins	+	+	+	+	+++	+++	+	+	+	+	+	+
Terpenoids	–	–	–	–	+++	++	++	–	+++	++	+++	+++
Glycosides	++	++	++	++	+++	+++	+++	+++	+	+	+	+

*AT* Assam green tea, *KW* Kashmir green tea, *IP* and *PN* Uttarakhand green tea

+++>++>+ indicates the intensity of color formation (difference in specific component conc. and – indicates no color (absence of specific component)

### Qualitative phytochemical screening

For the qualitative phytochemical screening of all four GT cultivars, standard procedures were followed to trace out the presence of the active principles, i.e., flavonoids, phenols, alkaloids, terpenoids, glycosides, tannins, anthocyanin, saponins, carbohydrates, and amino acid (Tariq and Reyaz 2012).

### Quantitative phytochemical screening

#### Determination of total phenolic content

Determination of total phenolic content was carried out using Folin–Ciocalteu reagent (FCR) assay by taking 20 µl of stock solution (1 mg/ml) of the extract dissolved in 80 µl of water and 500 µl of Folin–Ciocalteu reagent. The solutions were mixed and incubated in dark at room temperature for 5 min. After 5 min, 400 µl of 7.5 % sodium carbonate (Na_2_CO_3_) solution was added and the mixture was further incubated in dark for 30 min at room temperature. The absorbance of all samples was measured at 765 nm using colorimeter. Gallic acid was used as standard for the calibration curve that is plotted at 0.0156, 00312, 0.0625, 0.125, 0.25, 0.5, and 1.0 mg/ml of concentration, respectively. Triplicate measurements were carried out and total phenolic content was expressed as milligram of gallic acid equivalents (GAE) per gram of samples (Khan et al. 2011).

#### Determination of total flavonoid content

The flavonoid content was estimated by taking 100 µl of the sample in a test tube containing 400 µl of distilled water and subsequently 30 µl of 5 % sodium nitrite solution was added. After 5 min, 30 µl of 10 % aluminium chloride was added and allowed to stand for 5 min, then 20 µl of 4 % sodium hydroxide was added and the volume was adjusted up to 1 ml with distilled water. The absorbance of the mixture at 510 nm was measured immediately. Hydrated catechins were used as standard for the calibration curve that is plotted at 0.0156, 00312, 0.0625, 0.125, 0.25, 0.5, and 1.0 mg/ml of concentration, respectively. Triplicate measurements were carried out and total phenolic content was expressed as milligram of catechin equivalents per gram of samples (Subhashini et al. 2010).

#### Determination of the free-radical scavenging activity by the 1,1-Diphenyl-2-picrylhydrazyl (DPPH) free-radical scavenging assay

DPPH assay stock solution was prepared by dissolving 24 mg DPPH with 100 ml methanol and then stored at −20 °C until needed. The working solution was obtained by mixing 10 ml stock solution with 45 ml methanol to obtain an absorbance of 1.10 ± 0.02 units at 515 nm using spectrophotometer. 100 µl of leaf extract solution was allowed to react with 1,900 µl of the DPPH solution for 2 h in the dark. Then the absorbance was measured at 515 nm. Ascorbic acid was used as standard for the preparation of calibration curve that is plotted at 0.0156, 00312, 0.0625, 0.125, 0.25, 0.5, and 1.0 mg/ml of concentration respectively. Triplicate measurements were carried out and the percentage scavenging effect was calculated as:scavenging rate (%) = [(A_0_ − A_1_)/A_0_] × 100, where *A*_0_ was the absorbance of the control (without extract) and *A*_1_ was the absorbance in the presence of the extract (Azzahra et al. 2012).

#### Purification of GT polyphenols

The GT extracts namely AT, IP, KW, and PN were initially partitioned with water/chloroform (1:1). Then the water phase was collected and the impurities associated with the chloroform phase were discarded. As a second partition, water/ethyl acetate (1:1) was used. Polyphenol compounds such as catechins, epigallocatechin, epicatechin gallate, and epicatechin expected to have moved into the ethyl acetate layer were collected and concentrated by rota vapour for their analysis (Kyung and Yinzhe 2006).

#### Analysis of GT phytoconstituents by HPLC

##### Reagents and standard preparation

HPLC grade water and acetonitrile were used for the analysis. The standard compound (−)-epigallocatechin gallate (EGCg) was obtained from Sigma Aldrich (Catalogue No. RM10179). The standard with varying concentrations (12, 10, 8, 4, and 2 mg/ml) in acetonitrile was used for the plotting of standard calibration curve.

##### Sample preparation

The ethyl acetate residue was used to detect the EGCg concentration among the *C. sinensis* cultivars. The purified GT residue of different cultivars (AT, KW, PN and IP) was dissolved in acetonitrile for the HPLC analysis. The analytical determination of GT phytochemicals was performed using Dionex HPLC using varian, Microsorb-MV 100-5 C18 (250 × 4.6 mm) reverse phase column fitted in thermostatic column Compartment TCC-100 oven. The detector used for the analysis was UVD340U detector. Millipore syringe filter (0.2 µm) was used for the filtration of purified samples and the standard. Mobile phase contained acetonitrile:water in the ratio of 70:30. Ambient temperature was maintained at 28 °C; while the flow rate of the sample was adjusted to 0.5 ml/min. Detection wavelength was set at 280 nm. As a standard, solution of EGCg in acetonitrile was used. Volume of standard and a purified sample to be injected was 5 µl with a run time of 12 min (Yuegang et al. 2000). Identification of the specific polyphenol was carried out on the basis of their retention time. Quantification of polyphenol was directly performed by HPLC UVD340U detector using regression curve obtained by plotting the absorbance versus concentration of series of dilutions of standard EGCg.

## Results

The qualitative estimation of phytoconstituents in GT cultivars indicated the presence of pytosterols, catechin tannins, alkaloids, flavonoids, terpenoids, glycosides, carbohydrates and amino acids while the analysis revealed the absence of anthocyanin and saponins (Table 1). Based on the color consistency, the methanolic and acetone extracts have comparatively shown higher presence of the glycosides, catecholic tannins, and flavonoids while the presence of terpenoids was estimated higher in acetone and aqueous extracts.

Overall, the acetone extract of *Camellia sinensis* var. *sinsensis* and *assamica* cultivars had showed higher qualitative and quantitative presence of phytoconstituents than methanolic and aqueous extract. The percentage yields for all prepared extracts from GT had been evaluated that ranges from 3.07 to 26.8 % (Table 2) with the maximum yield depicted in acetone extract of Assam GT.

**Table 2:** The nature and color of crude extracts of green tea cultivars and their yield

Type of extract	Solvent	Green tea cultivars	Nature and color of extract	% yield
Crude	Methanolic	AT	Powdered, dark brown	4.00
		KW	Coal tar form, black	7.33
		PN	Lustrous thick, black	12
		IP	Lustrous thick, dark green	14.6
Crude	Acetone	AT	Thick, viscous, dark brown	26.8
		KW	Particulate, dark green	5.06
		PN	Particulate, dark green	9.00
		IP	Oily particulate, dark green	3.83
Crude extract	Aqueous	AT	Liquid, dark brown	3.07
		KW	Liquid, dark brown	7.33
		PN	Liquid, dark brown	12.00
		IP	Liquid, dark brown	14.6

Percentage yield = Weight of the sample extract obtained (g) × 100/Weight of the powdered sampled used (g)

**Fig. 3:**
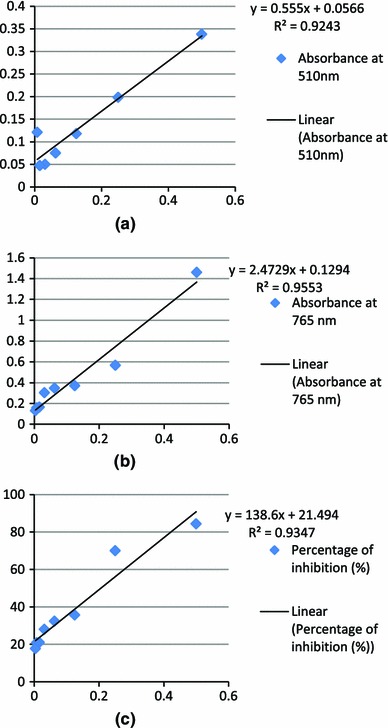
**a** Standard curve of catechin hydrate as a reference for total flavonoid content. **b** Standard curve of gallic acid as a reference for total phenolic content. **c** Standard curve for DPPH free radical scavenging activity

**Fig. 4:**
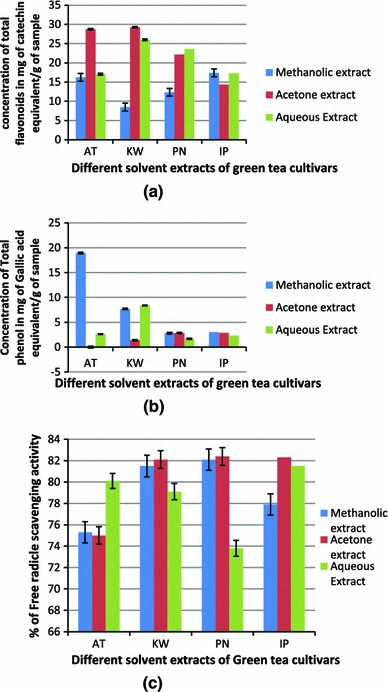
**a** Comparative analysis total flavonoids in mg of catechin equivalent/g of sample among different extracts of green tea cultivars. **b** Comparative analysis total phenolic content in mg of Gallic equivalent/g of sample among different extracts of green tea cultivars. **c** Comparative analysis of DPPH free radical scavenging activity among different extracts of green tea cultivars

Antioxidant activity, total phenol content and total flavonoid content were analyzed and their results were expressed as mean ± standard deviation. The flavonoids content in different solvent extracts of GT cultivars has been reported as catechin equivalents by reference to standard curve. It was estimated highest in acetone extract of GT cultivars with a concentration of 29.25 ± 0.015 mg of catechin equivalent/g of sample in Kashmir GT followed by aqueous extract with a concentration of 23.6 ± 0.017 mg of catechin equivalent/g of sample in PN GT extract (*P* ≥ 0.05) (Fig. 3a). The least flavonoids content 8.5 ± 0.003 mg of catechin equivalent/g of sample was calculated in methanolic extract of Kashmir GT (Table 3; Figs. 3a, 4a).

**Table 3:** Quantitative estimation of total flavonoids in aqueous and solvent extracts of different green tea cultivars

Extracts	Total flavonoids in green tea cultivars (mg of catechin equivalent/g of sample)			
	AT	KW	IP	PN
Methanolic	16.25 ± 0.030	8.5 ± 0.003	17.4 ± 0.003	12.35 ± 0.013
Acetone	28.75 ± 0.010	29.25 ± 0.015	14.35 ± 0.019	22.15 ± 0.005
Aqueous	17.05 ± 0.007	26.0 ± 0.077	17.3 ± 0.0007	23.6 ± 0.017

*AT* Assam green tea, *KW* Kashmir green tea, *IP* and *PN* Uttarakhand green tea

Significant difference in total phenolic content was observed among the investigated GT cultivars (*P* ≥ 0.05) (Fig. 3b). Methanolic extract of AT sample with a concentration of 18.32 ± 0.357 mg of gallic acid equivalent/g of sample depicted the highest concentration with reference to standard curve (Table 4; Figs. 3b, 4b).

**Table 4:** Quantitative estimation of total phenols in aqueous and solvent extracts of different green tea cultivars

Extracts	Total phenols in green tea cultivars (mg of gallic acid equivalent/g of sample)			
	AT	KW	IP	PN
Methanolic	18.32 ± 0.357	7.68 ± 0.165	3.02 ± 0.007	2.81 ± 0.142
Acetone	0.79 ± 0.020	1.4 ± 0.061	2.86 ± 0.074	2.84 ± 0.028
Aqueous	2.62 ± 0.101	8.41 ± 0.208	1.68 ± 0.0499	2.34 ± 0.038

*AT* Assam green tea, *KW* Kashmir green tea, *IP* and *PN* Uttarakhand green tea

There was significant difference in the antioxidant potentials of different extracts of GT cultivars (*P* ≥ 0.05) which represents the variation in percentage of oxidant scavenging capacity as performed by DPPH free radical scavenging assay (Table 5; Figs. 3c, 4c). It was found to be significantly greater in acetone extract of PN, KW and IP GT sample i.e., between 82.4 ± 0.004 and 82.3 ± 0.005 %. The least free radical scavenging activated was estimated to be 73.80 ± 0.152 % in aqueous extract of PN sample.

**Table 5:** Quantitative estimation of free radical scavenging activity by DPPH assay in aqueous and solvent extracts of different green tea cultivars

Extracts	Free radical scavenging activity in Green tea cultivars (% inhibition of DDPH radical)			
	AT	KW	IP	PN
Methanolic	75.30 ± 0.011	81.50 ± 0.002	77.90 ± 0.002	82.10 ± 0.016
Acetone	75.00 ± 0.053	82.10 ± 0.001	82.30 ± 0.0005	82.40 ± 0.004
Aqueous	80.10 ± 0.003	79.10 ± 0.003	81.50 ± 0.005	73.80 ± 0.152

*AT* Assam green tea, *KW* Kashmir green tea, *IP* and *PN* Uttarakhand green tea

**Fig. 5:**
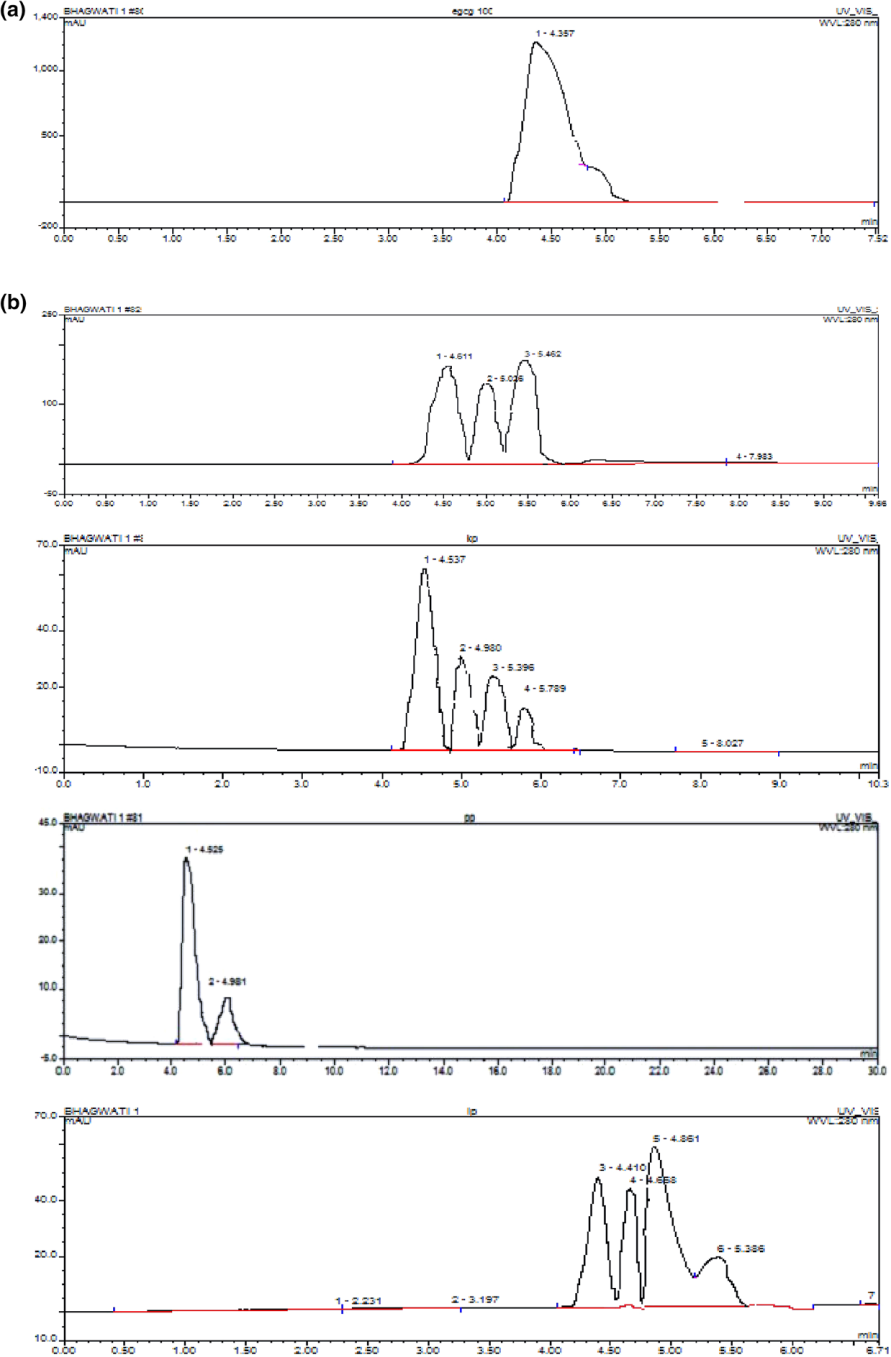
**a** HPLC chromatogram of standard EGCg. **b** HPLC chromatogram of purified residues of AT, KW, PN and IP extract

**Fig. 6:**
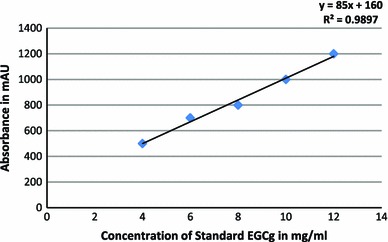
Standard calibration curve of Epigallocatechin gallate (EGCg)

**Fig. 7:**
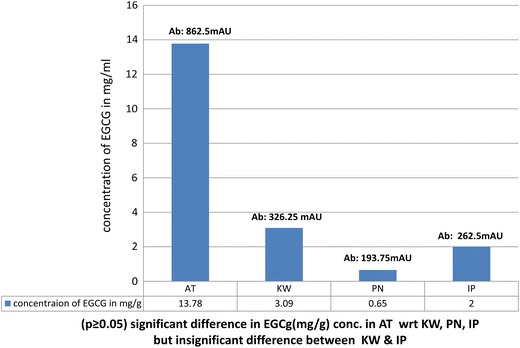
Concentration and absorbance (280 nm) of EGCG among GT cultivars

The HPLC calibration curve revealed the presence of EGCg based on the retention time of identified peaks compared with standard (4.457 min) (Fig. 5a, b). The concentration of EGCg in purified residues of GT cultivars was estimated in the range of 0.65 ± 1.76 mg/g of EGCg to 13.78 ± 3.535 mg/g of EGCg with approximately 71 % percentage of EGCg in the purified residue of AT. The regression line expressed as correlation coefficient was linear (*r*^2^ = 0.9897) built with standard EGCg (Figs. 6, 7).

Bases on Student’s *t* test, (*P* ≥ 0.05), a significant difference in the concentration of epigallocatechin gallate was depicted among Assam, Kashmir and Uttarakhand GT. However, there was insignificant difference in EGCg concentration between the GT cultivarscollected from Dehradun district.

## Discussion

Previously, it has been found that GT possesses tannins, steroids, flavonoids, and alkaloids that have medicinal value as well as food value (Tariq and Reyaz 2012). The multipotency of GT is due to the presence of an active component namely catechin, a flavonoid that serves in antimicrobial defence mechanism, high antioxidant property, prevention against molecular damage in cancer, protection against cardiovascular damage, diabetes, etc. (Anand et al. 2012). There is a positive and highly significant relationship between the phenolics and flavonoids with antioxidant activity of GT (Hajimahmoodi et al. 2008). A plethora of evidence suggests strong antioxidant potentials of tea catechins in suppressing the production of excess free radicals which at the molecular level help prevent cellular damage from certain oxidation reactions in the body (Sabhapondit et al. 2012). The total phenolic, flavonoids, and antioxidants activity are the parameters depicting the quality of tea according to their biological properties (Azzahra et al. 2012). Total catechin content could be used to indicate the quality potential of tea, with high content being related to high quality (Obanda et al. 1997). To ascertain diversity, careful study of secondary metabolites, especially those which are major contributors to quality, is essential.

Solvents, such as methanol, ethanol, acetone, ethyl acetate, and their combinations have been used for the extraction of phenolics from plant materials, often with different proportions of water. Selecting the right solvent affects the amount and rate of polyphenols extracted (Xu and Chang 2007). In particular, methanol has been generally found to be more efficient in extraction of lower molecular weight polyphenols while the higher molecular weight flavanols are better extracted with aqueous acetone (Metivier et al. 1980; Labarbe et al. 1999; Prior et al. 2001; Guyot et al. 2001).

As per the preliminary observation, the GT cultivars showed the presence of phytoconstituents with varying percentage yield. The percentage yields of methanolic, acetone and aqueous extracts are different from one plant to another. These differences might be explained due to the differences in the nature of the secondary plant metabolism and their solubility in different solvents (Al-Younis and Abdulla 2009).

Kashmir (KW) and Assam GT (AT) cultivars have shown maximum flavonoids and phenolic contents in their respective acetonic and methanolic extracts where as higher free radical scavenging activity was determined among all the extracted cultivars under investigation. The present study suggests that the free radical scavenging activity of the cultivars may be due to the presence of antioxidants, i.e., phenolic and flavonoids compounds as detected by the phytochemical estimation. In our initial study, separation and characterization of the catechins among the purified GT extracts by HPLC suggests the remarkable difference in the concentration of varying catechins that can be attributed to the differences in the geographical distribution of the cultivars.

## Conclusion

The phytochemical analysis of GT cultivars from Assam, Kashmir, and Uttarakhand region revealed the presence of terpenoids, tannins, alkaloids, phytosterols, glycosides, flavonoids, amino acid, and carbohydrates. The present comprehensive study demonstrates a marked influence of geographical location and the prevalent environmental specification (both agronomic and pedoclimatic) on the phytochemical characteristics of the GT cultivars. Owing to the significant interaction of the environment and catechin biosynthesis, the regional quality of GT cultivars can be enhanced by improvising the present expressions of the target genes characterized for catechin synthesis and hence will represent the regional cultivars as a marked herbal product with high nutraceutical application. Thus, further work is required to investigate these parameters to evaluate the diversity among the tea cultivars. Also, screening and characterization of the purified phytoconstituents by Prep-HPLC is essential to study the therapeutic potentials of GT and to confer it as a potent candidate for herbal drug development.

## Abbreviations

EGCg: Epigallocatechin gallate
GT: Green tea
AT: Assam green tea
KW: Kashmir green tea
IP, PN: Uttarakhand green tea
GAE: Gallic acid equivalent
DPPH: 1,1-Diphenyl-2-picrylhydrazyl
